# Digital Emotion Detection, Privacy, and the Law

**DOI:** 10.1007/s13347-025-00895-4

**Published:** 2025-05-27

**Authors:** Leonhard Menges, Eva Weber-Guskar

**Affiliations:** 1 Department of Philosophy, Faculty of Social Sciences, Paris Lodron University Salzburg, Franziskanergasse 1, 5020 Salzburg, Austria; 2 Institute of Philosophy I, Faculty for Philosophy and Science of Education, Ruhr-University Bochum, Universitätsstr. 150, 44801 Bochum, Germany

**Keywords:** Privacy, Right to privacy, Emotion recognition, Emotion detection, Law, Ethics of AI

## Abstract

Intuitively, it seems reasonable to prefer that not everyone knows about all our emotions, for example, who we are in love with, who we are angry with, and what we are ashamed of. Moreover, prominent examples in the philosophical discussion of privacy include emotions. Finally, empirical studies show that a significant number of people in the UK and US are uncomfortable with digital emotion detection. In light of this, it may be surprising to learn that current data protection laws in Europe, which are designed to protect privacy, do not specifically address data about emotions. Understanding and discussing this incongruity is the subject of this paper. We will argue for two main claims: first, that anonymous emotion data does not need special legal protection, and second, that there are very good moral reasons to provide non-anonymous emotion data with special legal protection.

When people are asked what they consider essential parts of their privacy, emotions often come up. There are at least some emotions that people prefer not everyone knows about: whom they are in love with or angry at, and what they are ashamed of. Prominent examples in the philosophical discussion about privacy also include emotions (e.g., Mainz, 2021; Matthews, 2008; Menges, 2022; Nissenbaum, 2004; Rachels, 1975; Reiman, 1976; Warren & Brandeis, 1984). Moreover, studies show that a considerable number of people in Great Britain and the US are uncomfortable with emotion detection in public settings generally or in certain situations, such as in the workplace (McStay, 2020; Roemmich et al., 2023). In light of these facts, it may be surprising to learn that current data protection laws in Europe, intended to protect privacy, do not specifically consider data about emotions. Understanding and discussing this incongruity is the topic of this paper.

The initial observation relates specifically to one aspect of the General Data Protection Regulation, or GDPR for short. This European Union regulation has been in effect since 2018, also impacting non-EU countries such as Switzerland, and is frequently praised as exemplary worldwide.^1^ It is explicitly motivated by the desire to protect individuals’ “privacy”.^2^ This aligns with the widely held view in society and in philosophical ethics that informational privacy is a good worth protecting. This concept of protection is fundamentally balanced with the goal of not unnecessarily obstructing the flow of data, as the flow of data is also very useful for many aspects of our lives.

So far, emotion data (understood in a broad sense that will be specified later) has not been specifically considered in this regulation.^3^ Given the rapid development of affective computing, which enables the digital and automated detection of emotions, this omission is remarkable, to say the least. The limited research literature on the subject also criticizes this oversight. For example, media scientist Andrew McStay writes that there is “a lacuna in critical literature on emotional AI and privacy, and in European law” (McStay, 2020, 4).

In this article we would like to clarify what types of emotion data are actually not or not sufficiently covered by the GDPR and discuss possible moral reasons for changing the latter. For this purpose, we will, after an explanation of the legal situation, give a brief insight into the foundations of the philosophy of emotions and of affective computing, in order to then discuss whether and if so, to what extent there are moral reasons to consider data on emotions as data worthy of legal protection. We will argue that there are both indirect as well as direct reasons to include non-anonymous emotion data in the group of so called “special categories” that deserve greater protection under the GDPR.^4^

## The Legal Situation

We begin with a brief overview of the legal situation. The reason why not all data about emotions are GDPR-relevant results from the definition of the data for which the regulation is issued. The relevant data is exclusively “personal data” meaning: “any information relating to an identified or identifiable natural person […]” (GDPR, Art. 4, 1). An identifiable natural person is in this context to be understood as.

one who can be identified, directly or indirectly, in particular by reference to an identifier such as a name, an identification number, location data, an online identifier or to one or more factors specific to the physical, physiological, genetic, mental, economic, cultural or social identity of that natural person (ibid.).

Data that is not related to an identified or identifiable person in this sense is considered anonymous data.

This appears to be a very *inclusive* understanding of relevant data. “Personal data” is not special data at all, such as particularly individual data like data about my own health situation, but all data that is known, or can be known, to belong to a particular person. The definition is *exclusive* only insofar as it excludes all *anonymized data*. For example, anyone may collect and process data about the weather without restriction – since this has nothing to do with persons at all. It is also permissible to collect data on how many university employees come to work by bike, but only if anonymity is preserved. Because in this case the data has to do with persons but does not refer to an identified or identifiable person. By contrast, the latter *is* the case, for example, when data is collected about which professor or employee uses the bicycle.^5^ Insofar as data about emotions belongs to persons, GDPR protections apply as soon as this data is personal in the sense of identifiability – it only does not apply if the data is anonymized.

But, crucially, the regulation distinguishes two levels of protection. For normal personal data, like data about which vehicle or route someone uses, the regulation just requires that data collection and processing take into account the following principles: lawfulness, fairness and transparency (traceability for the data subject), purpose limitation (not arbitrary), data minimization, accuracy, storage limitation, integrity and confidentiality, and accountability (GDPR, Art. 5). Some of these principles (transparency, data minimization, storage limitation, accountability) are usually fulfilled when it comes to analog, spontaneous, individual observations, because in these cases you can roughly estimate who and how many people see you where, and the scope and the storage of the data is generally limited. But the principles can easily be infringed when the observation is done by digital means (especially, when they are in the hands of powerful actors like big companies or the state). This can happen without notice of the observed persons much more easily than in the past. Furthermore, the situation is sharpened due to scaling, a fundamental feature of digitization: tens of thousands of data units can be collected in a matter of seconds and they can be analyzed, stored, and subsequently processed and used almost as quickly. Therefore, these principles, which are legally established and monitored for compliance, already provide a certain degree of protection.

The GDPR provides a *higher* level of protection for so-called “special categories” of personal data as defined above. For these special categories a general ban on collection and processing applies, unless there are certain conditions that justify exceptions. The reasons for these exceptions are more specific and demanding than those that justify the processing of data in the normal category. To illustrate, processing of special category data generally requires the explicit consent of the subject. Processing is allowed without consent only if it serves to protect vital interests and the person is physically or legally unable to give their consent (GDPR Art. 9,2 a and c). Normal personal data may be processed if it is vital while the consent of the subject is irrelevant. The special categories include:

personal data revealing racial or ethnic origin, political opinions, religious or philosophical beliefs, or trade union membership, as well as [...] genetic data, biometric data uniquely identifying a natural person, data concerning health or data concerning a natural person’s sex life or sexual orientation (GDPR Art. 9,1).

A natural question to ask is why, exactly, such data is taken to deserve special protection under the GDPR. An answer can be found in the Recitals (GDPR Recital 51): personal data are “by their nature, particularly sensitive in relation to fundamental rights and freedoms.” Therefore, Recital 51 continues, personal data “merit specific protection as the context of their processing could create significant risks to the fundamental rights and freedoms.” The core idea seems to be this: given what we know about the world, information about one’s racial and ethnic origin, political opinion, and so on can be used particularly easily by others to violate one’s fundamental rights and freedoms. In order to reduce this risk, the reasoning seems to go, such data is given special legal protection. As you can see, emotion data is not included in the special categories.

Starting from the common intuition that emotions are essential parts of a person’s privacy, we will scrutinize in the following if there are good moral reasons to include emotion data in the GDPR with a special focus on the possible threats of automated emotion detection. In doing so we will first clarify what emotions are and how far they can be detected by automated systems before subsequently developing the normative discussion.

## Theoretical Foundations

### Philosophy of Emotions

In the philosophy of emotions, authors distinguish between different types of states and processes within a subject that have phenomenal-qualitative content – that is, that feel a certain way.^6^ Emotions are one subcategory of such feelings, alongside others like bodily sensations and moods. One of the most fundamental disagreements in theories of emotion concerns whether or not emotions themselves contain a cognitive element. The latter claim was famously made in the strongest sense by William James. In his view, emotions are the feeling of bodily changes: “We feel sorry because we cry […] we [do not] cry because we are sorry” (James, 1884, 190). Although there is an ongoing discussion about how to correctly interpret James as a non-cognitivist (Reisenzein & Stephan, 2014), some scholars still defend this idea today (Prinz, 2004; Whiting, 2011). But the majority of current philosophical theories argue that emotions include some sort of cognitive element, because otherwise one cannot explain the intentionality of emotions. We are angry or sad *about* something, we are afraid *of* something, we are happy *about* something. Emotions are not only a hedonic experience, but such an experience in relation to something. And this structure is best explained by attributing some kind of cognitive content to the emotion which is then specified differently in the different theories of emotion, compared to a judgment (de Sousa, 1979; Nussbaum, 2001), a perception (Tappolet, 2000), an attitude (Deonna & Terroni, 2012), or by the new category of intentional feeling (Goldie, 2000; Helm, 2002). We will follow this insight and assume a kind of cognitive strand of emotion theory for the following considerations. But in general, everything we will say would probably also be plausible from the perspective of a non-cognitive theory of emotions because these theories do not deny any connection between emotions, beliefs, and values. They just do not see the cognitive aspects as an essential part of emotion. Some of the arguments we will make would then be somewhat indirect, but would not lose their general validity.

In short, we take emotions to be mental-bodily states and/or processes through which we react evaluatively to events in the world. They are directed toward something specific in the world, which they represent in some way, and to that extent they are intentional. As such, emotions differ, on the one hand, from mere bodily sensations, such as an itch, which are not world-related; and, on the other hand, they differ from moods, which are intentional in a different, more comprehensive way – in melancholy or euphoria, for instance, no single event but the whole world appears as dark and depressing or as bright and light, respectively. Typical examples of emotions include grief, joy, fear, anger, and disgust. Emotions are thus complex phenomena, characterized by phenomenal and intentional aspects, but beyond that also entail physical, motivational, and expressive aspects. They are closely related to moods because emotions can lead to moods, and moods are dispositions that support the emergence of emotions (see, e.g., Price, 2006). Grief, for example, is a hedonic negative emotion, related to death or separation, at any rate a loss, often accompanied by reduced bodily tension, crying, and withdrawal tendencies and that can lead to melancholy.^7^ This philosophical understanding of emotions and related phenomena also sets the groundwork for a discussion of what is known as “emotion detection” in the field of affective computing.

### Digital Emotion Detection as a Subfield of Affective Computing

Affective computing is a strand of AI research in which several sorts of computer systems are developed that can detect, evoke, or simulate emotions (Picard, 1997). Pioneer Rosalind Picard uses the term “affective” interchangeably with “emotional”, but ultimately means what we have described as being essential to “feelings”, namely anything that phenomenally-hedonically affects a subject (Picard, 1995, 5). The term “emotion recognition” has become established, but we prefer to use the term “emotion detection” in order to avoid the epistemologically demanding term “recognition”. It is also important to note that much of what is referred to as “emotion detection” does not only encompass emotions or moods in the narrow sense we described above, but also includes other types of feelings or states, such as tiredness and concentration (commonly used in in-cabin monitoring to alert drivers) or even purely physiological states, like those preceding an epileptic seizure. In this paper we will focus on the detection of current emotions and moods in the sense described above because these intuitively seem to be especially related to privacy concerns because of their specific personal nature.

Computers, bots, and robots using such software are meant to detect signs of emotions in humans, based on a variety of data, including what are thought to be visual expressions of emotion in the face or auditory expressions in the voice, or biomarkers, i.e., via physiological states such as skin conductance or heart rate. We will focus on the first type of emotion detection, on the visual one, for three reasons: First, based on a categorical theory, such visual detection is in principle apt to identify emotion types whereas technologies like those referring to physiological states are based on a dimensional theory and can only detect degrees of valence and arousal^8^; second, visual detection can be used from some distance without the targeted person knowing about it, whereas for physiological detection you need a wristband or something similar attached; third, it is technically already one of the most advanced and, accordingly, already quite widely used type of emotion detection (see García-Hernández et al., 2024, 8).

Digital visual emotion detection is largely based on the original basic emotion theory of Paul Ekman and Wallace Friesen (Ekman, 1982, 1992; Ekman & Friesen, 1978) and its further developments (see e.g. el Kaliouby, 2005; Picard, 1997). These approaches have in common that they are based on two theses in particular: (1) There are some clearly categorizable (and universally applied) emotion types such as joy, anger, fear, disgust, grief. (2) There are specific, externally detectable (and equally universally valid) bodily expressions for these emotion types: certain configurations of activated facial muscles, such as those we know as smiling, for example, as a sign of joy; or widened eyes as an expression of fear. The standard method was for some time that emotion detection systems first measure the movement of the muscles in the face and then provide a statistical number about what emotion is present. The Facial Action Coding System (FACS) developed by Ekman and Friesen divides the face into muscle units and their contraction possibilities, so-called action units, and assigns numbers to them (Ekman & Friesen, 1978). Specific compositions of the numbers are then assigned to specific emotion types (e.g., joy = 6 + 12; fear = 1 + 2 + 4 + 5 + 20 + 26). Meanwhile, affective computing also uses machine learning methods, that is, systems that are not programmed in reference to certain muscle contractions but that learn to identify signs of emotions in pictures and films displaying faces (and also posture and movements) after having been trained with labeled examples of pictures exhibiting signs of emotions (for a recent survey of the state of the art see Younis et al., 2024).

The universality claim of the basic emotion theory as well as the idea that there are any biological fingerprints of emotion types have been seriously contested (Barrett et al., 2019). There is not enough empirical evidence for a reliable and specific correlation between expressive displays and emotional states. We all know from everyday life that we do not always feel joy when we smile, and we do not always smile when we feel joy. We smile sometimes also when we are embarrassed about something or when we want to sell something. And we may have experienced subtle joy without any sign of a smile on our face. Another crucial issue that has to be kept in mind regarding the potential performance and power of emotion detection systems is that they, as such, only refer to the type of an emotion, not to the content of an emotion (Weber-Guskar, 2023). They point to joy, anger, fear etc., but cannot detect what the joy, anger, or fear is about. This has to be added by considering the context, but the same context can provide reasons for different types of emotion depending on the dispositions (values, preferences, aims, and autobiography) of the individual person.

Despite these general limitations of emotion detection systems, there are already many real-world applications in use. Furthermore, researchers mostly agree that there is some relation between physiological signs in the face, voice, gestures, and gait and experienced emotions (see cf. Calvo et al., 2015). It seems only a question of time until technology will advance and make more accurate detection possible. The most promising accounts are multi-modal approaches (Barrett et al., 2019, 49–50), combining expression detection with physiological measurements and a consideration of the situation. We think it is important to discuss the normative issues concerning emotion detection even before fully accurate technology is developed.

With this background, we can now proceed to the normative discussion of our topic, examining whether there are strong moral reasons for why digital emotion data should receive greater protection under the GDPR than it currently does. Of course, it has always been possible to detect emotions and moods from people’s facial expressions in public. But as we mentioned before, automated digital emotion detection is vastly different because of its ubiquity (cameras are on, even if no one is there), scale and the many ways to store and process the gathered data. For example, there is already a camera installed in the billboard at Piccadilly Circus in London, connected to a system that is said to detect emotions from the faces of passers-by(Cheshire, 2017; Bakir et al., 2022). Some supermarkets use similar technology for targeted marketing (Strathmann, 2017) or theft prevention (Blain, 2019). Companies are provided systems for emotion detection in order to evaluate reactions to presentations on conferences or fairs,^9^ and there have been scientific studies measuring the emotional reactions to big political events like an election campaign evening (McDuff et al., 2013).

## Should Emotion Data be Legally Protected?

Recall that the GDPR distinguishes four types of data:

1. Data that does not concern a person, that is not legally protected, such as data about the weather.
2. Data that concerns anonymous persons, such as data about how many students use a bike to go to university, which is also not protected.
3. Data that concerns an identified person (“personal data”), such as data about a specific student using a bike to go to university. This data may only be used if the principles of lawfulness, fairness, transparency, purpose limitation, data minimization, accuracy, storage limitation, integrity, confidentiality, and accountability are respected.
4. Personal data about racial or ethnic origin, political opinions, religious or philosophical beliefs, union membership, genetic or biometric data uniquely identifying a natural person (that is “hard” in contrast to “soft” biometrics), data about health, sex, or sexual orientation that concerns an identifiable or identified person – that is, the “special categories of personal data.” Such data may not be collected and processed unless certain conditions justifying exceptions apply, including the consent of the data subject, the pursuit of vital interests of the same or another natural person, or public interest in public health.

Depending on how we use the expression “emotion data”, such data is, by definition, about persons (and not about non-human animals, for example). Therefore, this data does not belong to category (1). In what follows, we will first discuss whether digital emotion data can be anonymous, such that it can fall under category (2). Second, non-anonymous emotion data clearly falls under category (3) and is, therefore, generally protected by the GDPR. However, we will discuss if in addition to that, non-anonymous emotion data should be treated as special data and would therefore fall under category (4). We will argue that, from a moral perspective, this should be the case.

### Emotion Data as Identifying Data

Some, stimulated by the intuition that emotions generally are especially private, might try to argue that there is never anonymous emotion data and, that therefore, emotion data should always be legally protected. There are two ways the thought can be spelled out. One might argue that to know someone’s current emotion *in depth* would be to know the person to a degree that would make it impossible for them to remain anonymous. The idea is that in order to know an emotion in depth one does not only need to know the type of the emotion (fear or joy) but also the concrete object of the emotion (what it is about) as well as the background conditions that make the concrete object the bearer of the formal object of an emotion (danger or a positive event), meaning the values, preferences, important memories and biographical information. These background conditions are at least partly constitutive of the identity of a person, or so one might argue.

Let us assume, for the sake of the argument, that knowing someone’s current emotion in depth requires the additional knowledge mentioned above and that having this additional knowledge involves identifying the person who has the emotion. It still does not follow that the automated emotion detection systems that are currently used reveal identifying information. These technologies measure signs and signals of emotions and provide statistical probabilities about what type of emotion the person has. However, they do not detect the additional information that is supposed to be necessary for knowing a person’s emotion in depth. For example, if the camera in the billboard at Piccadilly Circus detects the facial expression of a passer-by and infers that the person is, say, angry, without gathering any other data about them, this emotion data would be anonymous. In other words, current emotion detection systems do not allow us to know about a person’s current emotion *in depth*, and therefore, there can be anonymous emotion data.

An alternative attempt to justify the intuition that emotions are inherently personal adopts a technological perspective. Some argue that emotion data that is captured via the face can be understood as uniquely identifying. Gremsl and Hödl, for example, contend:

From our view it would […] be conceivable to define emotional data in terms of data protection law as personal data on emotional characteristics or states of a natural person obtained by means of special technical procedures, which *enable or confirm the unique identification of this natural person* (Gremsl & Hödl, 2022, 166, italics added).

If the relevant technology is such that emotion data enables unique identification, then there is no such thing as anonymous emotion data. Then, according to the standards of the GDPR, emotion data never falls under category (1) or (2) such that the collection of such data would always demand compliance with the principles of lawfulness, fairness, transparency, and so on. Moreover, if emotion data is uniquely identifying biometric data – like a fingerprint – it would even fall under category (4). Then, according to the principles of the GDPR, there should be a general prohibition on the processing of emotion data (with certain exceptions).

The line of thinking we have just presented relies on a problematic assumption. It says that the emotion detection procedures under consideration capture *an individual face* such that the person is identifiable. In fact, however, these systems are typically designed in such a way that they only detect that there is *some face*.^10^ To illustrate, compare a picture of you with a bright smile and a picture of an emoji with a bright smile. The first is clearly identifying, the second is not. Emotion detection systems are typically designed only to detect faces in analogy to the emoji and to analyze what emotional signs they express. That is, the face is scanned and measured to the extent necessary to detect a face at all and to determine the parts of the face – i.e., which pixels of the digital image belong to the eyes, the nose, or the mouth. The details that are needed to identify whose face it is, such as the distance between the eyes, are not determined. The system behind the camera in the billboard records muscle contractions and deduces from them whether a person is angry, sad, happy, and so on. Such data about emotion types are not identifying, because we share them with billions of other humans.

It can be objected that this data can be used for re-identification with the help of other software programs and data sets. This is plausibly true. However, the GDPR currently excludes overly elaborate ways of re-identification from its definition of identifiability.^11^ If one were to include any kind of identifiability, no matter how elaborate, the distinction between personal and non-personal would become virtually obsolete. This is because numerous studies on the re-identifiability of data sets of a certain size suggest that complete anonymization is nearly impossible in sufficiently large data sets (Jordan, 2022, 62, referring to Sweeney 2000; Solove & Schwartz, 2011). Thus, practically all digitally collected data about people would be personal data and would therefore be protected under the GDPR. Whether one should welcome or reject this implication is a question that we cannot answer here. Instead, we draw the following intermediate and conditional conclusion: If it makes sense to distinguish between digitally collected anonymous and personal data about people at all, then it is possible to design emotion detection systems in such a way that they only gather anonymous emotion data. In this case, emotion data from digital detection systems is not inherently identifying and it cannot be assumed that all such data would automatically receive legal protection under the GDPR. In what follows, we assume that it does make sense to distinguish between personal and anonymous digitally collected data.

### Emotion Data as a Special Category

Let us take a closer look at non-anonymous, or personal, emotion data. All personal data is legally protected by the GDPR. Some personal data has a special status, such as data about religious or political beliefs, sexual preferences, or health. The question we will focus on now is this: Should emotion data fall under this category? We will argue that from a moral perspective it should. We will present two primary arguments for this. The first is straightforward: Some emotion data is itself data about constitutive features of health, sexual orientation, and political opinion. We argue that insofar it is morally justified that data about health, sexual orientation, and so on, should have a special legal status – and we will argue in 3.2.2 that it is – then some emotion data should have the same legal status. The second is more indirect: The best moral arguments for providing special legal protection to data about ethnic origin, political opinion, religious beliefs, health, sex, and sexual orientation also suggest that emotion data should be provided special protection. We begin with the direct argument. But before that, we want to stress that we are concerned with *moral* reasons for legally protecting emotion data. We are therefore deliberately leaving it open whether there are stronger non-moral – perhaps legal, pragmatic, or procedural – considerations that speak against this.

#### The Direct Argument

A person’s emotions play a crucial part in constituting the person’s mental health. Take depression as an example. Two of depression’s core symptoms are a depressive mood often accompanied by emotions of anxiety, sadness, guilt, or irritability as well as anhedonia, which is the lack of joy and interest in things that one used to enjoy.^12^ Thus, part of what makes it the case that a person is depressed is that the person has certain moods or does not have certain emotions in certain situations. If a person did not suffer from a consistently low mood, for example, their mental health would be different. And if one is aware of her persistent low mood, repeated sadness, and lack of joy, one gains insight into an important aspect of her mind and overall mental health. There are already AI systems used to diagnose mental health issues like depression, where signs of emotion in the face play a role among other features (Mao et al., 2023). An even clearer example here is phobia. People with arachnophobia, for example, respond to the representation of spiders with persistent and excessive fear.^13^ Learning about a person’s tendency to respond with excessive fear to pictures of spiders is a way of learning about the person’s mental health. More generally, insofar as emotion detection systems successfully detect a person’s tendency to sadness or fear in certain situations they detect a constitutive feature of the person’s mental health.

One may reply that information about constitutive features of a person’s health is not itself information about a person’s health and that only the latter should be protected. In this case, the information that a person is depressed should be protected, but not the information that her mood is persistently low, for example. However, the idea that information about constitutive features of a person’s health status does not need special protection is independently implausible. It implies, for example, that while the information that a person has an ischemic stroke should have special protection, the information that the blood supply to parts of her brain is decreased – which is constitutive of such a stroke – does not require special protection. This seems to be an unacceptable conclusion. We therefore suggest that information about constitutive features of a person’s health should have the same protection as the information about their health status itself. More generally, insofar it is morally justified that mental health data should have special legal protection – as we will suggest below – then some personal emotion data should also have this protection.^14^ It would be morally arbitrary to provide special protection to the former but not the latter.

Something similar is true for sexual orientation, which can be defined by referring to emotional tendencies. According to the American Psychological Association, “sexual orientation” refers “to an enduring pattern of *emotional*, romantic, and/or sexual attractions to men, women, or both sexes.”^15^ Imagine that a person reliably responds with arousal to seeing billboards showing people of a specific gender in sexually provocative poses. If an emotion detection system detects this then it identifies part of what constitutes this person’s sexual orientation. Insofar it is morally justified that information about sexual orientation should have a special legal status according to GDPR – see Section 3.2.2 – then information about certain emotions should have the same legal status.

The case is a bit more complicated when it comes to a person’s political opinion. If political opinion is defined in terms of purely cognitive beliefs about political issues and if one rejects cognitivism about emotions – the view that emotions entail some sort of cognitive, belief-like element – then one cannot infer a person’s political opinions from knowing the person’s emotions. However, we see no good reason to assume such a narrow understanding of what a political opinion is and we assume, as explained at the beginning, that the many other authors that argue for a cognitive theory of emotions, are on the right track. It therefore seems more plausible that “political opinion” should be understood as including desires about how the world should be (e.g., that a certain candidate should win the election), and emotions (e.g., fear that a candidate will destroy democracy should they be elected, or anger about the candidates past actions). On this broader understanding of “political opinion”, some emotions will be political opinions in the relevant sense, such as fearing that a candidate will be elected, joy about the election of another candidate, or anger about a certain policy. If an emotion detection system identifies that a person reliably responds with, for example, fear,^16^ anger, and disgust to seeing billboards of a specific candidate then it detects the person’s political opinion, according to a plausible understanding of “political opinion”. Insofar it is morally justified that data about political opinions should be protected by the GDPR the same should be true for data about the relevant emotions.

To sum up, we have argued that some emotion data is data about constitutive features of health, sexual orientation, and political opinion. On the assumption that it is morally justified that the GDPR assigns special legal status to data about health, sexual orientation, and political opinion, it would be morally arbitrary not to assign the same status to the relevant emotion data. In what follows, we will, among other things, argue that it is morally justified that data about health, sexual orientation, and political opinion should have a special legal protection.

#### The Indirect Argument

The indirect argument to the conclusion that emotion data should be a special category under the GDPR says that the best moral arguments for giving special protection to data about health, ethnic origin, political beliefs, sexual orientation, and so on also suggest that emotion data should be provided special protection. Of course, the group of special categories as it exists in the GDPR is less the result of systematic moral reasoning than a reference to other important EU legal documents, such as the European Convention on Human Rights and the constitutions of the individual states of the Union, which themselves contain norms that have evolved historically. We believe, however, that it would be conducive to the legal regulation if it were to strive for the best systematic coherence regarding fundamental moral assessments and norms.

Here is our proposal in a nutshell: Given how our world is, when information about ethnic origin, sexual orientation, health and so on is private, some of our fundamental interests are served. If we have a fundamental interest in something that does not conflict with the fundamental interests of others, then we have, other things being equal, a strong moral right to that thing. We therefore have a strong moral right to privacy regarding these kinds of information. That we have a strong moral right to privacy regarding such data is the best moral justification for providing this data special legal status.

The line of thinking we have just presented is not innocent. However, its assumptions seem very plausible to us and are widely – but of course not universally – accepted. First, we assume that our privacy regarding, say, our health is diminished insofar as other people learn about our health situation against our will. This is consistent with most major theories of privacy that are currently discussed.^17^ Second, that information about, say, our health is private serves some of our fundamental interests. We will say more about this below. But it seems plausible that it is good for me to keep my tendency to depression private towards a potential future employer, for example. Third, we assume a broad version of the interest theory of moral rights, which holds that the purpose of moral rights is to protect certain important interests and that this purpose justifies the existence of specific moral rights. Obviously, this is a substantial premise (Wenar, 2020). But it fits nicely with many recent justifications of privacy rights (Marmor, 2015; Rössler, 2017; Véliz, 2022, 2024, ch. 10; Menges, 2024). Fourth, we assume that some – but of course not all – moral rights are so important that they should be legally protected and that the importance of the moral right provides the best moral justification for the legal protection. For example, the best moral justification for our legal right to physical integrity is that we have such a strong moral right to physical integrity. And we have such a strong moral right because it is in our fundamental interest to not be physically harmed.

Unfortunately, we do not have the space to argue for all these claims – it would take us several books. Our point is more modest. It is this: If one accepts this rough picture of the moral right to privacy and its connection to legal protection, then one should, from a moral perspective, also assign special legal status to emotion data. Let us elaborate.

A fundamental interest that is protected by the right to privacy is our interest in not becoming the victims of unjust discrimination. This interest is protected when, in our world, information about health, sexual orientation, religious beliefs, and so on is private, which is why we have a strong moral right to privacy regarding these kinds of information. And, therefore, these kinds of information deserve special legal protection (see, e.g. Munch, 2021; Véliz, 2024, ch. 7). We will argue that the same is true for information about people’s emotions.

That our world is such that information about a person’s religious or political views, sexual orientation, health, union membership and so on, can be used to discriminate against this person is, sadly, so plausible that we can take it for granted. What seems to need more justification is the claim that the same is true for information about one’s emotions. However, there is a good reason to believe this. Recall that emotions are, by definition, evaluative responses to an imagined or real event in the world. That is, by having an emotion we represent an aspect of the world evaluatively. Very roughly, grief, anger, fear, and so on share that they evaluate something as negative. Joy, relief, pride, and so on evaluate something as positive. This is the emotion’s *valence*. When we know how people evaluate their surroundings, we already know a lot about them and can make many reasonable hypotheses about them. To take up the former examples again, a person’s political and religious beliefs and their sexual orientation are partly constituted by how the person evaluates aspects of the world. Thus, when we know that some people reliably evaluate pictures of a certain politician or of people with a certain gender in provocative poses as positive then one can use this information to discriminate against them. Similarly, knowing that some people are constantly in the grip of sadness is knowledge about a constitutive feature of their mental health which allows us, again, to discriminate against them. More generally, as we can be the victims of unjust discrimination because of what others know about our emotions, emotion data deserve special protection.

A second fundamental interest that is protected by the moral right to privacy is our ability to shape personal relationships (see, e.g., Fried, 1984; Rachels, 1975; Marmor, 2015; Menges, 2024). It is of fundamental importance for us to be able to distinguish between, say, close friends and distant colleagues. One important way to make this distinction is by sharing different kinds of information. We can foster friendships by sharing personal information about health, religion, sexual orientation, and so on. And we can keep distance by making sure that our colleagues or students do not learn about these matters. This way of shaping personal relationships requires privacy. If all data about me is known by everyone, then I cannot shape personal relationships in this way. Thus, my right to privacy serves my fundamental interest in being able to shape personal relationships. As we have seen, information that belongs to the special category is exactly the kind of information that we can use to foster intimate friendships by sharing it and to foster social distance by keeping it private.

So far, this is a standard argument for the right to privacy. What we add is the very plausible idea that information about our emotions is also important for building intimacy or social distance (see also Fried, 1984). One way of forming intimacy is to share what we enjoy, fear, love, or what we find disgusting or shameful. Imagine that friends tell you that they feel disgust when visiting their sick grandparents, and that they are ashamed because of their own disgust. This is an understandable and burdensome situation for your friends, and sharing such a burden is a way of shaping an intimate friendship. Not sharing information of this kind towards, say, one’s colleagues is, relatedly, an important way of keeping professional distance. This shows that – like information about religious views, sexual orientation, health, and so on – information about our emotions is important for shaping personal relationships.

Thus, just as we have a fundamental interest in keeping, say, health data private because it is important for shaping relationships, we also have such an interest in keeping emotion data private. As the first interest justifies a moral right to privacy which then morally justifies special legal protection for health data, the same should be true for emotion data.

To sum up, the indirect argument looks at the best moral justification for giving special legal status to the data that in fact does have a special legal status in the GDPR. Based on some plausible – but, of course, not self-evident – assumptions we have presented two justifications: this data deserves special legal protection because this protects our fundamental interest in not becoming victims of unjust discrimination and in being able to shape personal relationships. We have argued that the same fundamental interests are protected if emotion data is assigned special legal status. Therefore, we conclude, there is good moral reason to assign emotion data the same kind of legal protection that is already assigned to data about health, sex, religious beliefs, and so on.

#### Defending the Indirect Argument

We will now discuss two objections raised against the argument presented above.^18^

Some may object that the indirect argument relies on the assumption that the point of the GDPR is to protect people’s informational privacy. However, they may continue, this is far from obvious. It has been observed that the word “privacy” is not mentioned once in the GDPR, while expressions like “data protection” are often used (Buckley et al., 2024; Leibovitz, 2018). This suggests that the GDPR is more about data protection than about privacy, the objection continues. If this is so, then we cannot assume that moral reasons for protecting informational privacy are relevant for the GDPR. Then, the indirect argument would not work.

Let us make two points in reply. First, even if the word “privacy” is not used in the GDPR itself, it is used in documents that explain its point for a broader audience, and it was used by the European Commission to motivate the need for GDPR in the first place.^19^ For example, the official website of the GDPR begins its main text with the claim that “[t]he General Data Protection Regulation (GDPR) is the toughest privacy and security law in the world” (EU, 2018). This shows that at least important representatives of the EU take the GDPR to protect informational privacy. Second, even if these representatives misunderstand the GDPR’s purpose and the GDPR is, in fact, not about protecting privacy but about data protection, the indirect argument works after making some non-substantial modifications. What we have said in the previous sub-section does not lose plausibility if one replaces each reference to informational privacy with a reference to data protection. The conclusion would still be that the best moral arguments for giving special protection to data about health, ethnic origin, political beliefs, sexual orientation, and so on also suggest that emotion data should be provided special protection.^20^

A second possible objection against the indirect argument is that it overgeneralizes: it implies that too many things fall in the special category. The indirect argument relies on the idea, the objection says, that every kind of personal information that is such that it can be used to discriminate against the person and that the person can use to shape inter-personal intimacy or distance should have special legal status. The objection continues that every kind of information can in principle be used for both purposes. Then, however, the indirect argument collapses because it is surely not true that every kind of information should have special legal status.

As a reply, the indirect argument does not need to assume that every kind of information that can in principle be used to discriminate against people and that people can use to shape intimacy or distance should have special legal protection. To see this, focus first on the legal perspective. The distinction between the special category and other personal information is drawn by reference to the *risk* that this kind of information is used in certain, for example discriminatory, ways (GDPR Recital 51). To illustrate, it is *in principle* possible to discriminate against students based on the information that they go to university by bike – imagine a dystopian authoritarian right-wing regime that regards the use of bicycles as subversive behavior. The risk that this will happen is, fortunately, very low in the current EU. Therefore, information about going to university by bike and similar kinds of information is currently not regarded as belonging to the special category. This is the legal perspective, but it corresponds to the moral perspective we adopt here. According to the most plausible version of the indirect argument, whether or not a kind of information is protected by a strong moral right to privacy depends on how likely it is that the relevant information can be used to discriminate against people, and how important it is to shape personal intimacy and distance (see Menges, 2024, sect. IV.4). The world we are living in is such that information about whether students go to university by bike is typically not used to discriminate against them or to shape intimacy or distance. Therefore, the best version of the indirect argument implies that this kind of information does not need special protection. And what is true for information about how people go to university is true for many kinds of information.

More generally, the indirect argument does not overgeneralize. Even if it is in principle possible to use every piece of information to discriminate against people, it does not follow that every piece of information deserves special protection. Only those kinds of information deserve special protection that are such that it is sufficiently likely that others can use it to discriminate against people and that people use it to shape intimacy or distance. And we think we have shown that it is sufficiently likely for information about emotions.

## Conclusion

The starting point of this paper was the conflict between the everyday idea that emotions are clearly private and the fact that the most important European legal regulation on data and privacy does not mention them even though computer systems are able to detect and process people’s emotions to a certain extent and with a certain degree of probability. We have argued for the following claims: first, on the currently used understanding of “anonymous,” emotion data can be anonymous data and such data does not need to be protected. Second, and more importantly, if the detection is not anonymous, then there is a strong moral reason to treat emotion data like data about health, sexual orientation, and religious beliefs and to provide such data not only regular, but even special legal protection. These consideration can also be seen as a justification for the recently established ban of emotion detection systems in the new EU AI Act and should even be helpful for further work on the legal document that might, in future, extend the areas for the ban.

## Data Availability

Not applicable.

## Footnotes

1 E.g., “The G.D.P.R. was heralded as the world's most comprehensive data privacy law when it was enacted, and championed as a model for the rest of the world to counter the data-hording practices of Facebook, Google and other internet giants.” https://www.nytimes.com/2021/09/02/business/facebook-whatsapp-privacy-fine.html?searchResultPosition=10

2 This can be seen for example on the landing page of the official webpage of the GDPR. https://eur-lex.europa.eu/LexUriServ/LexUriServ.do?uri=COM:2012:0009:FIN:DE:PDF.

3 “It is not clear to what extent EU data protection law, particularly the GDPR, is fit for purpose when applied to the processing of emotional data based on AC [affective computing] approaches” (Häuselmann, 2021, 247).

4 Meanwhile, in 2024, a new European regulation has been implementd, the EU AI Act. This is the first legal document that prohibits placing on the market, putting into service for this specific purpose, or use of AI systems to infer emotions of a natural person in the areas of workplace and education institutions except in cases where the use of the AI system is intended for medical or safety reason (see article 5 (f)); furthermore, emotion detection systems are more generally categorizised as high-risk systems, being therewith subject to strict rules of transparency etc. (ANNEX III). Our paper offers a rationale for classifying emotion detection as a high-risk activity as well as a philosophical justification for a ban on emotion detection software at least in certain areas.

5 On the Internet, data is considered personal, even if it is not linked to a name or a job. An identification number such as an IP address is sufficient in this regard, because a person can in principle be identified by it.

6 See for this entire section Scarantino and de Sousa 2021 Summer Edition, and more specifically Goldie, 2000, Weber-Guskar 2009.

7 While there is agreement across different theoretical approaches on at least a core group of phenomena that belong to emotions, the use of the term “affect” is more diverse. Recently, affect studies following postmodern approaches have developed a concept of affect under which carriers of affects are no longer subjects themselves, but affects exist as something *between* people, animals, or things (Massumi, 2002; Slaby, 2019).

8 Categorical theories of emotion imply that there are several clearly distinct categories (or types) of emotions (like fear, joy, anger, grief, disgust) whereas dimensional theories of emotions distinguish gradually between different emotional states on the two dimensions of valence and arousal (where joy is more positive and of high arousal and grief negative and of low arousal).

9 See for example: https://xpai.io/

10 See for example Bishay et al. 2023 where three steps are distinguished: face tracking (not “recognition”), AU (action unit) detection, metrics estimation (prediction of an emotion).

11 This is mentioned e.g. at Häuselmann, 2021, 248. He refers to Case C-582/14, Breyer v Federal Republic of Germany [2016] ECR I-779 (ECLI:EU:C:2016:779) para 46. For a way to prevent such re-identification technically, see Narula et al. 2020.

12 See standard diagnosis of depression, cf. at https://www.nimh.nih.gov/health/publications/depression

13 See ICD 11 6B03, https://icd.who.int/browse/2024-01/mms/en#239513569

14 Note, again, that the detection system itself only detects the movements of a face, which are then labeled as being the expression of an emotion. It does not detect what the emotion is about. E.g., the system tells us that people are scared, but not that they are scared of spiders. However, we are assuming here and in the following that there can be situation in which the context allows the users of the system to make reasonable hypotheses about the object of the emotion. This is the case, for example, when the system detects people’s emotions in front of billboards showing spiders. Of course, it is not 100% certain because the person could at that exact moment be imagining or remembering a totally different scary object, but these should be considered exceptional cases.

15 https://www.apa.org/topics/lgbtq/orientation

16 We assume that there are possibilities for detecting a certain reliability of responses without losing the condition of anonymity; this could be done, for example, by presenting the same politician at different spots in a public space within the span of a few minutes.

17 See, e.g., recent access (Macnish, 2018; Véliz, 2024, ch. 5) and control (Marmor, 2015; Menges, 2022) theories of privacy. For an overview see Rössler & DeCew (2023).

18 Thanks to an anonymous referee for pressing us on this.

19 European Commission (2012): https://eur-lex.europa.eu/LexUriServ/LexUriServ.do?uri=COM:2012:0009:FIN:DE:PDF.

20 To illustrate, consider a modified version of our main argument in a nutshell: Given how our world is, when *data* about ethnic origin, sexual orientation, health and so on is *protected*, some of our fundamental interests are served. Moreover, and most importantly, when data about our emotions is protected, then some of our fundamental interests are served. If we have a fundamental interest in something that does not conflict with the fundamental interests of others, then we have, other things being equal, a strong moral right to that thing. We therefore have a strong moral right *that these kinds of data be protected*. That we have a strong moral right to *protection* regarding such *data* is the best moral justification for providing such data special legal status.
